# Synergistic activity of Hsp90 inhibitors and anticancer agents in pancreatic cancer cell cultures

**DOI:** 10.1038/s41598-019-52652-1

**Published:** 2019-11-07

**Authors:** Simonas Daunys, Daumantas Matulis, Vilma Petrikaitė

**Affiliations:** 1 Faculty of Pharmacy, Lithuanian University of Health Sciences, Sukileliu Av. 13, LT-50162 Kaunas, Lithuania; 2 Institute of Biotechnology, Life Sciences Center, Vilnius University, Sauletekio Av. 7, LT-10257 Vilnius, Lithuania; 3 Laboratory of Drug Targets Histopathology, Institute of Cardiology, Lithuanian University of Health Sciences, Sukileliu Av. 13, LT-50162 Kaunas, Lithuania

**Keywords:** Mechanisms of disease, Cancer models

## Abstract

Heat shock protein 90 (Hsp90) is a widely investigated target for anticancer therapy. The experimental Hsp90 inhibitors ICPD47 and ICPD62 demonstrated anticancer activity against colorectal, osteosarcoma and cervical cancer cell lines. However, their anticancer activity has not been investigated against pancreatic cancer cell lines yet, and there are no data about synergistic activity of these compounds in combination with clinically used anticancer agents. Pancreatic cancer cell lines, MIA PaCa-2 and PANC-1 were exposed to ICPD47 and ICPD62 alone and in combinations with antimetabolites gemcitabine (GEM), 5-fluorouracil (5-FU) and topoisomerase inhibitor doxorubicin (DOX). Effects on cell viability were determined by MTT assay. The synergistic activity was evaluated using Chou-Talalay method. Also, 3D cell cultures were formed using 3D Bioprinting method and the activity of each compound and their combinations was examined by measuring the size change of spheroids. The strongest synergistic activities were determined in combinations using all ratios of ICPD47 with GEM and ICPD62 with GEM in MIA PaCa-2 cell line (combination index <0.5). The combinations of ICPD47 with 5-FU and ICPD47 with GEM in a ratio of 1:5 showed the greatest effect on tumour spheroid growth in both cell lines. The ICPD47 in combination with mild hyperthermia showed significant results, where the *EC*_50_ value in PANC-1 cell line dropped from 4.04 ± 0.046 to 1.68 ± 0.004 µM. The ICPD47 and ICPD62 under the same conditions could act synergistically with GEM, 5-FU and DOX and is worth of further investigations, and studies of synergistic effect is a promising path for more efficient anticancer therapies.

## Introduction

Pancreatic cancer is one of the most aggressive and deadliest cancer types. After diagnosis, the patients have a 5-year survival rate of only 8.5%. Such a low survival rate is mainly because the disease is diagnosed at late stages and it progresses fast to lymphatic system and local organs^1,2^. In addition, the increasing resistance of pancreatic cancer to available chemotherapy is a serious problem.

There are seventeen registered drugs for pancreatic cancer chemotherapy, but statistics show that the current treatment is not effective. Recent studies show, that the resistance for 5-fluorouracil (5-FU) and gemcitabine (GEM) is increasing^3^. Therefore, there is a huge need for novel therapeutic agents that could act on multiple targets and enhance the success of pancreatic cancer therapy.

Heat shock protein 90 (Hsp90) is a chaperone protein of 90 kDa. Hsp90 is required for the folding and stability of proteins, which are critical for cell growth, differentiation and survival^4,5^. Since this molecule regulates multiple signalling cascades, the inhibition of Hsp90 has a suppressive effect on many client proteins and biochemical pathways^5^. Because of this characteristic and the fact that in cancer tissues Hsp90 is expressed in higher levels, the inhibition of Hsp90 can overcome the redundancies of signals and drug resistance in many cancers^5^. Recent scientific and clinical data show that Hsp90 inhibitors strongly affect cancer cell survival and using them in combinations with anticancer agents, it is possible to postpone cell resistance to chemotherapy^6,7^.

Combinatorial chemotherapy is often used for the treatment of cancers. Synergistic effect of different drugs in combinations can increase its therapeutic effect, decrease dosage and toxicity, compared to individual drugs^8^. To determine the combination effect, the Chou-Talalay method is often used, when the combination index (*CI*) is calculated. This method is the most accurate to evaluate the type of combinatorial effect of different compounds. *CI* values below 1 (*CI* < 1) describe synergistic effect, values equal to 1 (*CI* = 1) describe additive effect, and values over 1 (*CI* > 1) show antagonistic effect of the compounds^8^.

There are many scientific manuscripts published on the combinations of Hsp90 inhibitors and anticancer agents in various cancers except for pancreatic cancer. Never the less, credible data on synergistic activity of Hsp90 inhibitors and anticancer agents are limited or falsely determined. The main mistakes in determining the combinatorial effects of the drugs are: (a) using statistical analysis and calculating *P* values, rather than Chou-Talalay method, when *CI* is calculated; (b) using randomly chosen concentrations of compounds in the combination instead of using constant *EC*_50_ ratios of compounds in the combination^8^. Some studies have shown that the expression of Hsp90 in pancreatic cancer tissue and in tissues adjacent to cancer is higher up to five fold, compared to healthy pancreatic tissue^9,10^. With this study, we wanted to obtain new fundamental insights on the effects of Hsp90 inhibitors in combinations with already used anticancer agents (GEM, 5-FU and DOX) against pancreatic cancer cell lines. Therefore, for the first time, we tried to determine the combination effect of experimental resorcinol-like Hsp90 inhibitors (ICPD47 and ICPD62) and clinically used anticancer agents in two-dimensional (2D) and three-dimensional (3D) models of different pancreatic cancer cell lines. In addition, we aimed to determine whether a mild hyperthermia could increase the activity of Hsp90 inhibitors.

## Results

### Hsp90 inhibitors inhibit viability of pancreatic cancer cells

**Figure 1:**
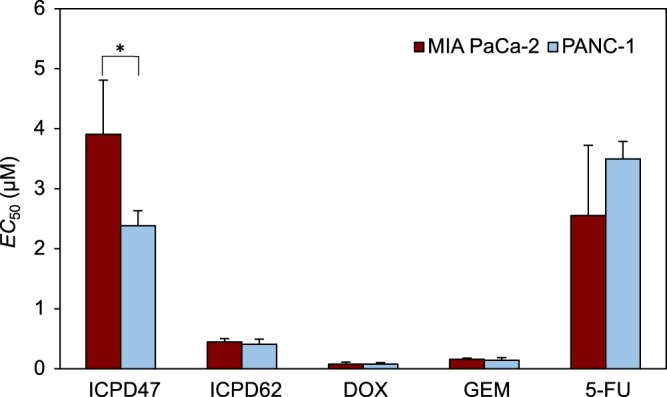
Effect of Hsp90 inhibitors and anticancer agents on cell viability (*p < 0.05, n = 3).

Hsp90 inhibitors and anticancer agents demonstrated different effects on cell viability (Fig. 1). All compounds inhibited cell viability in both pancreatic cancer cell lines. Results showed that, ICPD47 inhibited the viability of PANC-1 cells almost 2 times stronger than MIA PaCa-2 cells (*p* < 0.05). The ICPD62 had higher effect on cell viability than ICPD47, almost nine times in MIA PaCa-2 cell line, half maximal effective concentration (*EC*_50_) values of ICPD62 and ICPD47 after 72 h were 0.45 ± 0.06 µM and 3.91 ± 0.25 µM. And in PANC-1 cell line ICPD62 inhibited cell viability six times stronger than ICPD47, as *EC*_50_ values of ICPD62 and ICPD47 after 72 h were 0.41 ± 0.08 µM and 2.38 ± 0.25 µM (Fig. 1). In the anticancer agents group, DOX had the greatest activity on cell viability (*EC*_50_ after 72 h on MIA PaCa-2 and PANC-1 cells were 0.08 ± 0.03 µM and 0.07 ± 0.03 µM, respectively).

**Figure 2:**
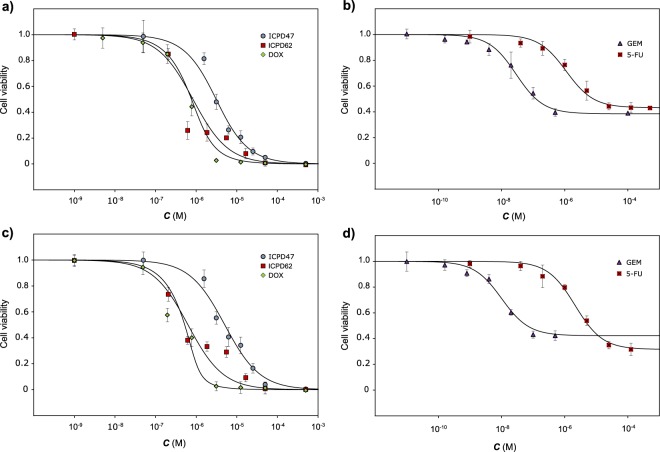
Activity of Hsp90 inhibitors and anticancer agents on pancreatic cancer cell viability. (**a,b**) – MIA PaCa-2 cell line, (**c,d**) – PANC-1 cell line.

Similar to DOX, both Hsp90 inhibitors showed cytotoxic activity, as they totally inhibited cell viability in higher concentrations (Fig. 2a,c). The GEM and 5-FU belong to the class of antimetabolites and reduced cell viability down to 40 and 30%, respectively (Fig. 2b,d).

### Combinations of Hsp90 inhibitors and anticancer agents lead to synergistic effects on cell viability

**Figure 3:**
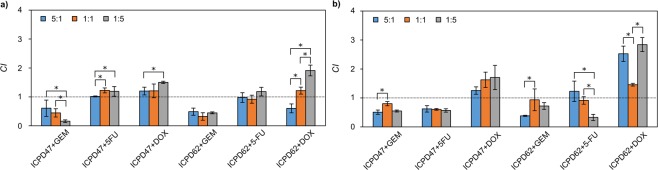
*CI* values of Hsp90 inhibitors and anticancer agents when *fa* = 0.5. (**a**) – MIA PaCa-2 cell line, (**b**) – PANC-1 cell line. *CI* < 1 synergistic activity, *CI* = 1 – additive effect, *CI* > 1 – antagonistic activity. *p < 0.05.

Most of the combinations exhibited a synergistic effect on both pancreatic cancer cell lines at some point. The strongest synergistic effects were determined using combinations of ICPD47 with GEM (*CI* value, when fraction affected (*fa*) equal to 0.5, was 0.16 ± 0.05) in ratio 1:5 in MIA PaCa-2 cell line (Fig. 3a), and ICPD62 with 5-FU in ratio 1:5 (*CI* value when *fa* = 0.5 was 0.33 ± 0.11) in PANC-1 cell line (Fig. 3b).

**Figure 4:**
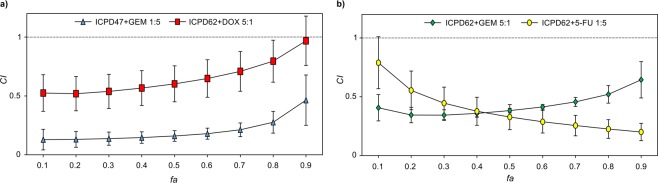
Strongest synergistic activities of Hsp90 inhibitors and anticancer agent combinations in: (**a)** - MIA PaCa-2 cell line, (**b)** - PANC-1.

The combinations of ICPD47 with GEM in ratio 1:5 and ICPD62 with DOX in a ratio of 5:1 showed synergistic effects (*CI* < 1) in all concentrations (*fa* range from 0 to 1) in MIA PaCa-2 cell line (Fig. 4a). In PANC-1 cell line the only combinations that acted synergistically were ICPD62 with GEM in ratio of 5:1, and ICPD62 with 5-FU in ratio of 1:5 (Fig. 4b).

**Figure 5:**
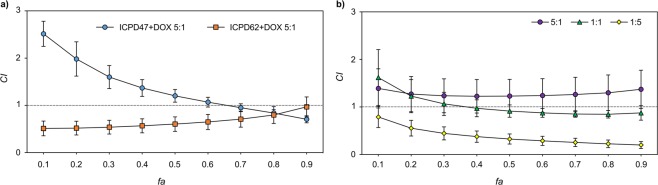
The dependence of combination effect under various conditions: (**a**) combination effect of ICPD47 and ICPD62 with DOX in 5:1 *EC*_50_ ratio in MIA PaCa-2 cell line; (**b**) combination effect of ICPD62 with 5-FU in different *EC*_50_ ratios in PANC-1 cell line.

In this project, we have determined that different Hsp90 inhibitors in combination with the same anticancer agent and in the same concentration ratios can have opposite effects on cell viability (Fig. 5a).

It was also determined that the combinations of same compounds but in different concentration ratios may have a different effect on cell viability (Fig. 5b).

### Hsp90 inhibitors and their combinations with anticancer agents affect the growth of pancreatic cancer cell spheroids

**Figure 6:**
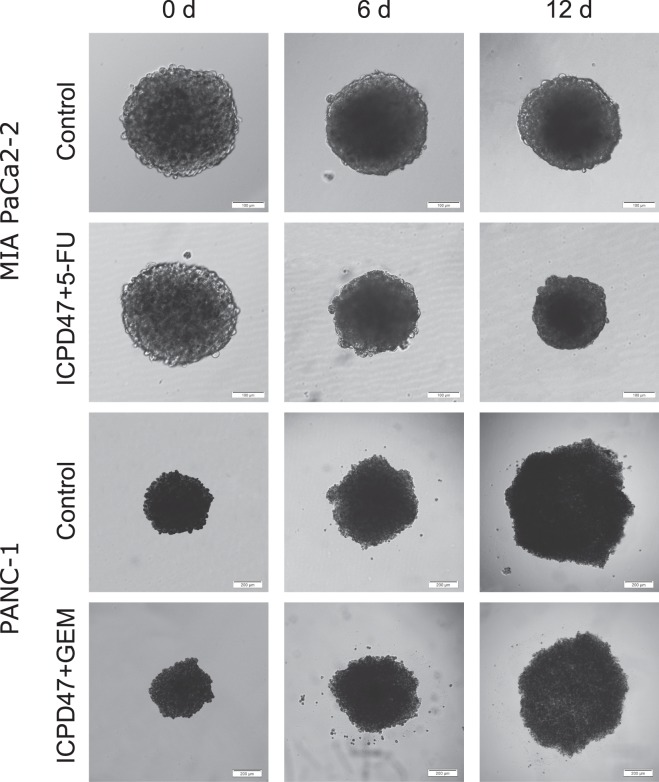
The effect of HSP90 inhibitor and anticancer agent combinations on MIA PaCa-2 (magnification 100×) and PANC-1 (magnification 40×) cells spheroids.

MIA PaCa-2 cells formed small compact spheroids (344 ± 8.4 μm in diameter), PANC-1 cells formed relatively small and compact spheroids (401 ± 13.8 μm in diameter) (Fig. 6). The effects of all combinations and separate compounds on pancreatic cancer spheroids can be found as Supplementary Table S1 for MIA PaCa-2 and Supplementary Table S2 for PANC-1 cell lines.

**Figure 7:**
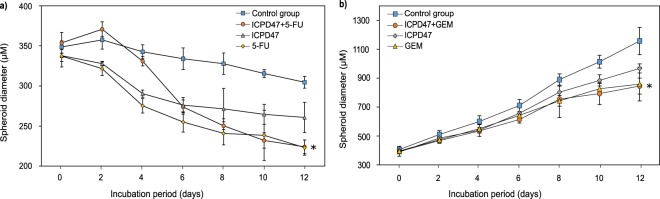
Hsp90 inhibitors, anticancer agent and their combination effect on spheroid growth: (**a**) – MIA PaCa-2 spheroids, (**b**) – PANC-1 spheroids (*p < 0.05; n = 8).

All compounds and their combinations reduced the size of MIA PaCa-2 spheroids. The strongest reduction was caused by the combination of ICPD47 with 5-FU in ratio of 1:5 and 5-FU alone. After 12 day exposure to the combination, the spheroid size reduced 1.6 times (*p* < 0.05), and 5-FU reduced the size of the spheroids by 1.5 times (*p* < 0.05) (Fig. 7a). However, the difference between the effects of the combination and 5-FU alone was not significant (*p* > 0.05). The least effective compounds were ICPD62 and DOX, as they reduced the size of the spheroid by 1.17 and 1.25 times respectively (Supplementary Figs S1 and S2,A).

The size of PANC-1 spheroids was increasing during an entire experiment. The partial dynamics of the PANC-1 spheroids growth can be found as Supplementary Fig. S1. The size of PANC-1 spheroids exposed to the combination of ICPD47 and GEM and GEM alone increased slower compared to control and other groups (Fig. 7b). After twelve days of incubation with this combination and GEM alone, the average diameter of spheroids (n = 8) was 1.37 (*p* < 0.05) and 1.35 (*p* < 0.05) times smaller compared to control group. However, the difference between the effects of the combination and GEM alone was not significant (*p* > 0.05). ICPD62, DOX and 5-FU had almost no effect on the growth of PANC-1 spheroids (Supplementary Fig. S2).

### Hyperthermia increases the effect of ICPD47

**Figure 8:**
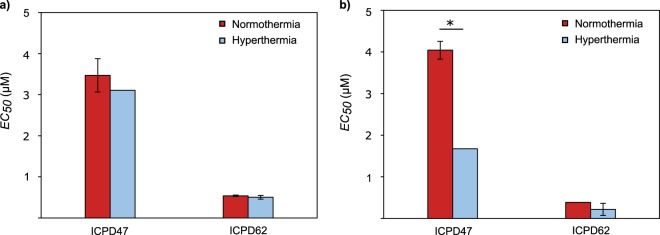
The effect of Hsp90 inhibitors and hyperthermia: (**a**) – MIA PaCa-2 cell line, (**b**) – PANC-1 cell line (*p < 0.05; n = 3).

ICPD47 in combination with hyperthermia had greater effect on cell viability than ICPD47 itself. *EC*_50_ value in PANC-1 cell lines decreased 2.4 times, from 4.04 ± 0.046 to 1.68 ± 0.004 µM (*p < *0.05) and in MIA PaCa-2 - 1.2 times, from 3.71 ± 0.01 to 2.48 ± 1.65 µM (*p* > 0.05) (Fig. 8a). ICPD62 in combination with hyperthermia also had a greater effect on cell viability than ICPD62 itself. *EC*_50_ value in MIA PaCa-2 cell line decreased 1.1 times from 0.49 ± 0.01 to 0.40 ± 0.03 µM), and in PANC-1 - 1.8 times from 0.39 ± 0.01 to 0.22 ± 0.02 µM, but the results were not of exceptional significance (*p* > 0.05) (Fig. 8b).

## Discussion

In our study, ICPD62 showed from 6 to 9 times greater effect on cell viability than ICPD47, in PANC-1 and MIA PaCa-2 cell lines, respectively. Similar results were demonstrated in Cikotiene *et al*. study, where ICPD62 showed almost 10 times higher activity than ICPD47 in U2OS and HeLa cell lines^11^. Such results can be explained by the differences in compound structure^12^.

The strongest synergistic activity was determined by using combinations of Hsp90 inhibitors with GEM and 5-FU. According to different studies of Fallahi-Sichani and Arlander, the Hsp90 inhibitors may slow down the cell metabolism, and therefore making them more sensitive to antimetabolites^13,14^. Also it is possible, that some antimetabolites, such as cisplatin, may inhibit Hsp90 by binding to its’ C-domain, and therefore using it in combination with Hsp90 inhibitors can enhance the inhibition of Hsp90^15^. Kaiser *et al*. in their study determined that DOX in combination with resorcinol-like Hsp90 inhibitor could have synergistic effect on cell viability, at higher concentrations of compounds when higher concentrations of compounds are used. However, due to variable conditions, such as incubation plan and time with individual compounds, the effect can vary from synergism to antagonism^16^.

In this study, it was determined that different Hsp90 inhibitors in combination with the same anticancer agent exhibiting the same *EC*_50_ ratio, can have different effect, even though, both inhibitors have the same mechanism of action. It is possible that such differences in action can be influenced by different structures and activities of both compounds. Lee *et al*. in their study determined synergistic activity of combinations of Hsp90 inhibitor NVP-AUY922 and 5-FU against gastric cancer cells^17^. Researchers explained that NVP-AUY922 inhibited the expression of thymidylate synthase (TS), which is the main reason of increased resistance of cancer cells to 5-FU. Therefore, decreased level of TS, makes gastric cancer cell more sensitive to 5-FU^17^. It is possible, that pancreatic cancer cells ICPD47 interacts with 5-FU in similar way. In different study, Liu *et al*. determined that 5-FU in combination with another Hsp90 inhibitor, SNX-2112, showed an antagonistic effect on human oesophageal cancer cell viability^4^. In their opinion, 5-FU could compete with SNX-2112 in binding to Hsp90 N-domain, or induced the cancer signal paths that were inhibited by SNX-2112.

To our knowledge, no studies have been conducted to evaluate combination effects of Hsp90 inhibitors and anticancer agents in pancreatic cancer spheroids. The combination of ICPD47 with 5-FU had a greater effect on MIA PaCa-2 spheroid growth than individual compounds (*p < *0.05). The ICDPD47 combination with GEM had greater effect on PANC-1 spheroid growth (*p < *0.05) than individual compounds. According to scientific literature, pancreatic cancer cell lines are different in genotype and phenotype, so their aggregation qualities and spheroid formation abilities are different. Therefore, it could determine different spheroid growth kinetics between MIA PaCa-2 and PANC-1 cell lines. Overall, the combinations of compounds had greater potency in 2D monolayer cultures than in 3D spheroid cultures. The diffusion of the compounds to inner layers of spheroid could be limited due to high density of the cells and the increased levels of structures of extracellular matrix, which makes a natural barrier similar in solid tumors, therefore only the outer layers could be affected^18^. Also, the genes related to increased resistance to anticancer agents could be more activated in spheroids than in monolayer cells^18,19^.

In our study, mild hyperthermia has increased the effect of ICPD47 in both pancreatic cancer lines. Similar results were shown in Ito *et al*. study, where hyperthermia of 43 °C increased the activity of Hsp90 inhibitor geldanamycin 1.8 times against human melanoma cells compared to the effect of hyperthermia and compound, separately^20^. Krawczyk *et al*. demonstrated that the combination of 17-DMAG and 43 °C temperature had greater activity on murine rabdomyosarcoma cell cultures than the compound and hyperthermia alone^21^. Different groups of scientists have also determined that Hsp90 inhibitors could increase the thermosensitivity of cancer cells and reduce the response to the heat shock^21,22^. This data shows that using Hsp90 inhibitors in combination with mild hyperthermia could be an interesting and successful approach in cancer therapy, though more thorough research is needed.

## Conclusion

In conclusion, the combinations of Hsp90 inhibitors and anticancer agents can have synergistic anticancer effect in pancreatic cancer cell lines under specific conditions. The most potent and promising combinations were ICPD47 with GEM, and ICPD47 with 5-FU, and are worth of further investigations.

## Materials and Methods

### Compounds

Hsp90 inhibitors ICPD47 and ICPD62 were synthesized at Vilnius University, Lithuania as previously described^11^. GEM was bought from Enzo (Tilst, Denmark), and DOX hydrochloride and 5-FU were purchased from Sigma-Aldrich Co. (St Louis, MO, USA).

### Cell cultures

Human pancreatic cancer cell lines MIA PaCa-2 and PANC-1 were obtained from the American Type Culture Collection (ATCC, Manassas, VA, USA). Human foreskin fibroblast cells HF were provided by prof. Santos A. Helder (University of Helsinki, Finland). All cell lines were cultured in Dulbecco’s Medium GlutaMAX (DMEM Glutamax) medium. The medium was supplemented with 10,000 U/mL penicillin, 10 mg/mL streptomycin, and 10% fetal bovine serum. The medium and supplements were purchased from Gibco (Carlsbad, CA, USA). Cells were maintained in humidified atmosphere containing 5% CO_2_ at 37 °C.

### Cell viability

Compound effect on cell viability was established using 3-(4,5-dimethylthiazol-2-yl)-2,5-diphenyltetrazolium bromide (MTT; Sigma-Aldrich Co., St Louis, MO, USA) assay. The MIA PaCa-2 and PANC-1 cells were seeded in 96-well plates in a volume of 100 µL (5,000 cells/well). After 24 h preincubation, the cells were treated with 100 µL of different concentrations of Hsp90 inhibitors and anticancer agents. The medium without cells was used as a positive control, and the medium with 0.25% DMSO (Sigma-Aldrich Co.) served as a negative control. After 72 h, the cells were incubated with 20 µL 5 mg/mL MTT solution (Sigma-Aldrich Co.) for 4 h under the same conditions. The absorbance was measured at wavelengths of 570 and 630 nm, and the *EC*_50_ (half-maximal effective concentration of a drug at which 50% of its maximum response is observed) of the compounds were calculated.

### The determination of combination index

The *EC*_50_ of each compound was determined as described previously. To evaluate the effect of combination treatment, different combinations of Hsp90 inhibitors and anticancer drugs in three constant ratios (5:1, 1:1, 1:5) of *EC*_50_ were prepared and serial dilutions were made. The combinations of the compounds were added to the 96-well cell plates with cells after 24 h preincubation and incubated for 72 h. After incubation the MTT assay was performed. To calculate the combination indices (*CI*), the CompuSyn software was used (Version 1.0; ComboSyn Inc., Paramus, NJ, USA) that is based of Chou-Talalay method^8^.

### Activity in spheroids

Spheroids were formed from MIA PaCa-2 and PANC-1 cells using 3D Bioprinting method^23^. The pancreatic cancer cells were mixed with human fibroblasts in a ratio of 1:1 in order to better represent the tumour microenvironment^24^. The cells were incubated with nanoparticles NanoShuttle (Nano3D Biosciences Inc., Houston, TX, USA) for 8 h. The cells were resuspended and seeded into ultra-low attachment 96-well plates in a volume of 100 µL (2,000 pancreatic cancer cells and 2,000 human fibroblasts per well). The plate was placed on magnetic drive and incubated in humidified atmosphere containing 5% CO_2_ at 37 °C for 72 hours until spheroids were formed. The formed spheroids were imaged using light microscopy (Olympus TH4-200). Then, the medium was replaced with the fresh one, containing different concentrations of separate compounds and their combinations in a ratio of 1:5 at the concentrations when *fa* was 0.5. The photographs of spheroids were taken every 48 h, using Olympus cellSens Standard 1.16 software (Olympus Corporation, Japan). The medium was replaced every 96 h^25^.

The effect of Hsp90 inhibitors, anticancer agents, and their combinations on spheroid growth was examined by measuring the size change of spheroids using ImageJ software (National institute of Health, USA).

### Hsp90 inhibitors and hyperthermia synergistic assay

The cell suspension was prepared and seeded as described previously in cell viability assay in two 96-well plates. After 24-hour preincubation, one plate was heated in a water bath at 43 °C temperature for 30 minutes. Then the cell viability assay (as described previously) was performed to establish *EC*_50_ values of single Hsp90 inhibitors and the combination of inhibitors and hyperthermia.

### Statistical analysis

Statistical analysis was performed using Microsoft Office Excel 2007 software (Microsoft Corporation, Redmond, WA, USA). All experiments were performed in at least triplicate independent measurements and the obtained values were reported as mean ± standard deviation. Student’s t-test was used and p-values were calculated. A value of *p* < 0.05 was considered as the level of significance.

### Ethical approval

This article does not contain any studies with human participants or animals performed by any of the authors.

### Informed consent

For this type of study, formal consent is not required.

## Supplementary information

Supplementary data
