# Supplementary material for: Synergistic activity of Hsp90 inhibitors and anticancer agents in pancreatic cancer cell cultures
Source: Sci Rep. 2019 Nov 7;9:16177. doi: 10.1038/s41598-019-52652-1 (PMC6838130; doi:10.1038/s41598-019-52652-1)
Supplement: Supplementary file 1 — Supplementary data [file 41598_2019_52652_MOESM1_ESM.docx]

**Synergistic activity of Hsp90 inhibitors and anticancer agents in pancreatic cancer cell cultures**

Simonas Daunys^1^, Daumantas Matulis^2^, Vilma Petrikaite^2,3^

^1^ Faculty of Pharmacy, Lithuanian University of Health Sciences, Sukileliu Ave. 13, LT-50162 Kaunas, Lithuania

^2^ Institute of Biotechnology, Life Sciences Center, Vilnius University, Sauletekio Av. 7, LT-10257 Vilnius, Lithuania

^3^ Laboratory of Drug Targets Histopathology, Institute of Cardiology, Lithuanian University of Health Sciences, Sukileliu Ave. 13, LT-50162 Kaunas, Lithuania

Corresponding author:

Tel: +370 686 29383

E-mail address: [VilmaPetrikaite@gmail.com](mailto:VilmaPetrikaite@gmail.com) (V. Petrikaitė)

ORCID

0000-0002-6178-6276 Daumantas Matulis

0000-0002-4106-5535 Vilma Petrikaitė

0000-0002-2371-9881 Simonas Daunys

**Table S1.** Average diameter (in µm) of MIA PaCa-2 spheroids during 12 days of incubation with combinations and separate compounds (n=8)

|  | **0 d.** | **SE** | **2 d.** | **SE** | **4 d.** | **SE** | **6 d.** | **SE** | **8 d.** | **SE** | **10 d.** | **SE** | **12 d.** | **SE** |
| --- | --- | --- | --- | --- | --- | --- | --- | --- | --- | --- | --- | --- | --- | --- |
| **Control** | 349 | 8 | 358 | 11 | 343 | 9 | 334 | 14 | 328 | 13 | 316 | 5 | 305 | 7 |
| **ICPD47+GEM** | 354 | 8 | 370 | 1 | 341 | 13 | 306 | 11 | 294 | 17 | 297 | 18 | 290 | 23 |
| **ICPD47 (0,22 µM)** | 335 | 9 | 327 | 8 | 303 | 11 | 288 | 9 | 287 | 17 | 281 | 16 | 279 | 7 |
| **GEM (0,04 µM)** | 341 | 18 | 344 | 25 | 334 | 11 | 307 | 11 | 302 | 13 | 297 | 14 | 296 | 26 |
| **ICPD47+5-FU** | 354 | 13 | 371 | 9 | 331 | 6 | 274 | 9 | 250 | 9 | 232 | 10 | 224 | 9 |
| **ICPD47 (1,42 µM)** | 338 | 7 | 328 | 3 | 290 | 4 | 276 | 9 | 271 | 26 | 265 | 12 | 261 | 19 |
| **5-FU (5,08 µM)** | 337 | 14 | 321 | 8 | 275 | 9 | 255 | 13 | 241 | 14 | 238 | 11 | 223 | 9 |
| **ICPD47+DOX** | 341 | 6 | 367 | 5 | 343 | 8 | 311 | 5 | 293 | 9 | 285 | 12 | 275 | 16 |
| **ICPD47 (1 µM)** | 341 | 13 | 363 | 7 | 328 | 13 | 298 | 11 | 283 | 12 | 271 | 13 | 264 | 17 |
| **DOX(0,1 µM)** | 334 | 3 | 326 | 5 | 319 | 9 | 301 | 3 | 293 | 18 | 289 | 11 | 286 | 10 |
| **ICPD62+GEM** | 357 | 10 | 363 | 10 | 329 | 16 | 301 | 14 | 287 | 22 | 282 | 28 | 282 | 29 |
| **ICPD62 (0,08 µM)** | 341 | 8 | 348 | 13 | 338 | 3 | 319 | 11 | 314 | 9 | 306 | 10 | 305 | 19 |
| **GEM (0,13 µM)** | 335 | 7 | 319 | 4 | 306 | 24 | 272 | 11 | 256 | 20 | 252 | 9 | 248 | 26 |
| **ICPD62+5-FU** | 358 | 10 | 371 | 10 | 347 | 6 | 310 | 9 | 288 | 7 | 269 | 8 | 260 | 11 |
| **ICPD62 (0,13 µM)** | 338 | 6 | 338 | 27 | 332 | 7 | 317 | 27 | 306 | 8 | 299 | 10 | 296 | 19 |
| **5-FU (3,57 µM)** | 335 | 4 | 327 | 3 | 286 | 9 | 287 | 27 | 265 | 6 | 253 | 13 | 249 | 14 |
| **ICPD62+DOX** | 350 | 6 | 373 | 5 | 364 | 9 | 345 | 8 | 331 | 9 | 310 | 7 | 298 | 13 |
| **ICPD62 (0,21 µM)** | 345 | 4 | 366 | 17 | 324 | 18 | 312 | 17 | 307 | 13 | 300 | 18 | 295 | 15 |
| **DOX (0,171 µM)** | 336 | 9 | 329 | 9 | 299 | 31 | 296 | 8 | 287 | 9 | 281 | 12 | 268 | 35 |

**Table S2.** Average diameter (in µm) of PANC-1 spheroids during 12 days of incubation with combinations and separate compounds (n=8)

|  | **0 d.** | **SE** | **2 d.** | **SE** | **4 d.** | **SE** | **6 d.** | **SE** | **8 d.** | **SE** | **10 d.** | **SE** | **12 d.** | **SE** |
| --- | --- | --- | --- | --- | --- | --- | --- | --- | --- | --- | --- | --- | --- | --- |
| **Control** | 406 | 23 | 510 | 27 | 600 | 40 | 711 | 41 | 891 | 39 | 1014 | 44 | 1157 | 64 |
| **ICPD47+GEM** | 393 | 16 | 468 | 19 | 535 | 16 | 615 | 25 | 755 | 128 | 794 | 76 | 846 | 55 |
| **ICPD47 (1,13 µM)** | 389 | 29 | 486 | 31 | 540 | 43 | 658 | 38 | 803 | 43 | 885 | 39 | 967 | 31 |
| **GEM (0,34 µM)** | 397 | 8 | 474 | 16 | 550 | 28 | 642 | 25 | 743 | 36 | 827 | 36 | 859 | 57 |
| **ICPD47+5-FU** | 415 | 37 | 508 | 39 | 577 | 25 | 682 | 38 | 825 | 32 | 952 | 40 | 965 | 292 |
| **ICPD47 (0,39 µM)** | 393 | 20 | 497 | 18 | 577 | 28 | 674 | 26 | 822 | 45 | 914 | 57 | 1007 | 58 |
| **5-FU (3,28 µM)** | 418 | 22 | 418 | 24 | 590 | 21 | 705 | 19 | 847 | 17 | 1005 | 31 | 1088 | 67 |
| **ICPD47+DOX** | 378 | 15 | 478 | 16 | 537 | 18 | 633 | 30 | 762 | 43 | 887 | 50 | 993 | 66 |
| **ICPD47 (1,06 µM)** | 383 | 21 | 496 | 18 | 622 | 36 | 688 | 34 | 863 | 33 | 944 | 28 | 997 | 28 |
| **DOX(0,17 µM)** | 411 | 26 | 502 | 21 | 568 | 31 | 676 | 41 | 833 | 52 | 972 | 51 | 1094 | 53 |
| **ICPD62+GEM** | 389 | 15 | 483 | 13 | 563 | 18 | 649 | 22 | 788 | 32 | 872 | 35 | 957 | 61 |
| **ICPD62 (0,14 µM)** | 398 | 9 | 509 | 14 | 595 | 33 | 709 | 28 | 878 | 69 | 1001 | 118 | 1125 | 222 |
| **GEM (0,24 µM)** | 415 | 19 | 500 | 11 | 577 | 34 | 681 | 30 | 812 | 48 | 931 | 60 | 1125 | 106 |
| **ICPD62+5-FU** | 398 | 16 | 488 | 27 | 580 | 20 | 687 | 22 | 852 | 38 | 966 | 46 | 1092 | 113 |
| **ICPD62 (0,04 µM)** | 432 | 26 | 530 | 27 | 622 | 41 | 730 | 39 | 896 | 52 | 989 | 60 | 1081 | 64 |
| **5-FU (1,61 µM)** | 402 | 17 | 458 | 24 | 638 | 23 | 744 | 16 | 895 | 17 | 1100 | 36 | 1138 | 57 |
| **ICPD62+DOX** | 386 | 21 | 483 | 20 | 554 | 16 | 671 | 29 | 831 | 33 | 961 | 53 | 1079 | 104 |
| **ICPD62 (0,29 µM)** | 393 | 12 | 491 | 22 | 588 | 26 | 689 | 25 | 869 | 48 | 985 | 54 | 1071 | 53 |
| **DOX (0,27 µM)** | 406 | 22 | 514 | 26 | 573 | 34 | 686 | 22 | 841 | 47 | 983 | 44 | 1082 | 74 |


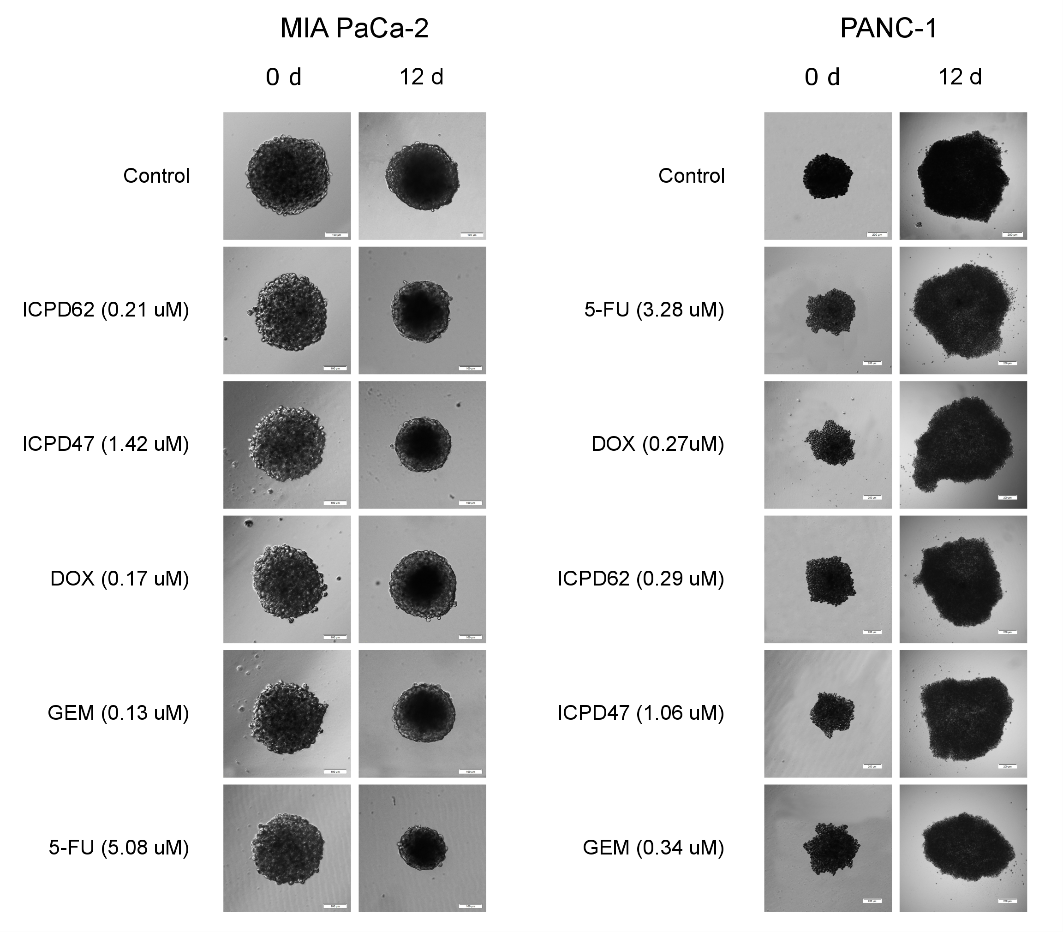


***Figure S1.*** *The comparison of the diameters of MIA PaCa-2 and PANC-1 spheroids, after 12 days of incubation with separate compounds (n=8)*

*
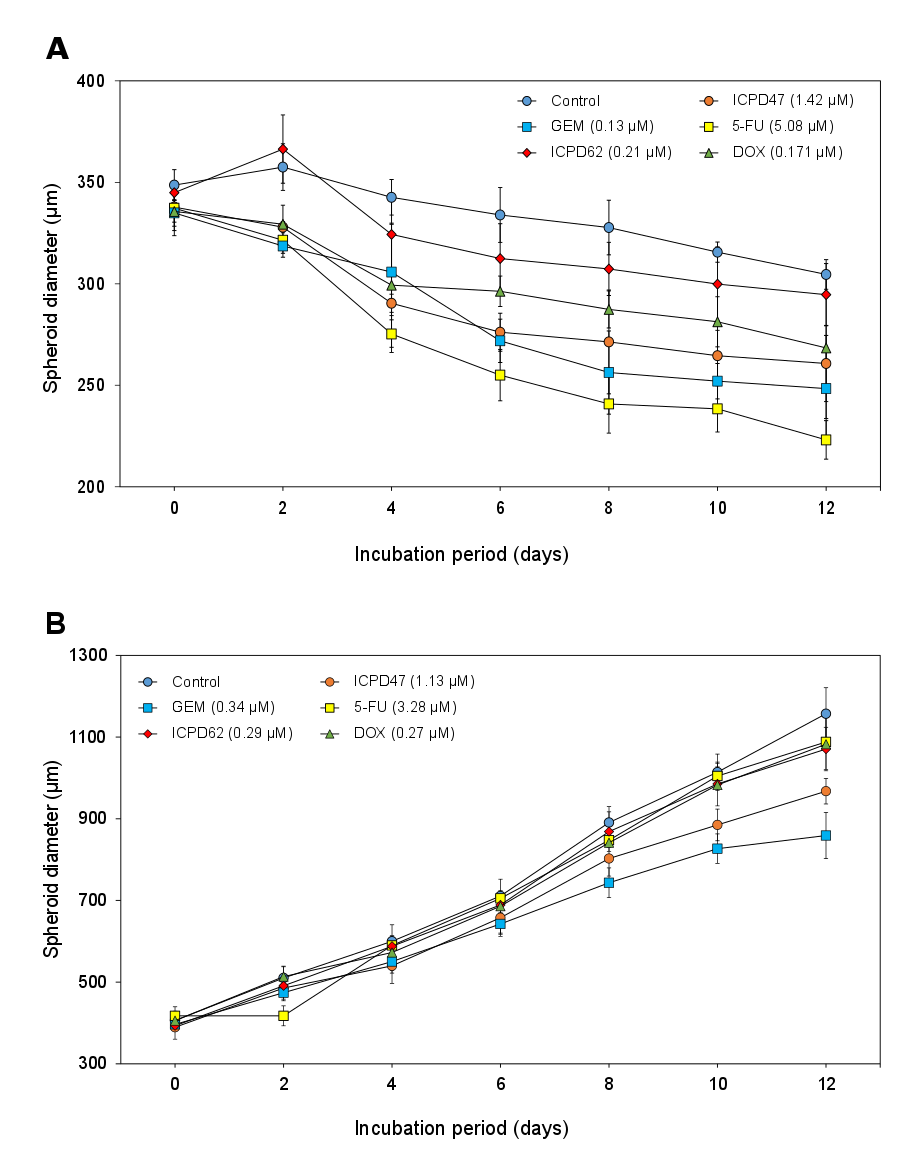
*

***Figure S2.*** *The effect of separate compounds on the growth of MIA PaCa-2 (A) and PANC-1 (B) spheroids (n=8, p < 0.05)*
